# Nanogels as target drug delivery systems in cancer therapy: A review of the last decade

**DOI:** 10.3389/fphar.2022.874510

**Published:** 2022-09-08

**Authors:** Anthony A. Attama, Petra O. Nnamani, Ozioma B. Onokala, Agatha A. Ugwu, Adaeze L. Onugwu

**Affiliations:** ^1^ Drug Delivery and Nanomedicine Research Group, Department of Pharmaceutics, University of Nigeria, Nsukka, Enugu, Nigeria; ^2^ Public Health and Environmental Sustainability Research Group, Department of Pharmaceutics, University of Nigeria, Nsukka, Enugu, Nigeria; ^3^ Institute for Drug-Herbal Medicines-Excipients Research and Development, University of Nigeria, Nsukka, Enugu, Nigeria

**Keywords:** nanoparticles, cancer therapy, targeted drug delivery, nanogels, future prospects

## Abstract

Cancer is an important cause of morbidity and mortality worldwide, irrespective of the level of human development. Globally, it was estimated that there were 19.3 million new cases of cancer and almost 10 million deaths from cancer in 2020. The importance of prevention, early detection as well as effective cancer therapies cannot be over-emphasized. One of the important strategies in cancer therapy is targeted drug delivery to the specific tumor sites. Nanogels are among the several drug delivery systems (DDS) being explored as potential candidates for targeted drug delivery in cancer therapy. Nanogels, which are new generation, versatile DDS with the possession of dual characteristics of hydrogels and nanoparticles have shown great potential as targeted DDS in cancer therapy. Nanogels are hydrogels with a three-dimensional (3D) tunable porous structure and a particle size in the nanometre range, from 20 to 200 nm. They have been visualized as ideal DDS with enormous drug loading capacity, and high stability. Nanogels can be modified to achieve active targeting and enhance drug accumulation in disease sites. They can be designed to be stimulus-responsive, and react to internal or external stimuli such as pH, temperature, light, redox, thus resulting in the controlled release of loaded drug. This prevents drug accumulation in non-target tissues and minimizes the side effects of the drug. Drugs with severe adverse effects, short circulation half-life, and easy degradability by enzymes, such as anti-cancer drugs, and proteins, are suitable for delivery by chemically cross-linked or physically assembled nanogel systems. This systematic review summarizes the evolution of nanogels for targeted drug delivery for cancer therapy over the last decade. On-going clinical trials and recent applications of nanogels as targeted DDS for cancer therapy will be discussed in detail. The review will be concluded with discussions on safety and regulatory considerations as well as future research prospects of nanogel-targeted drug delivery for cancer therapy.

## 1 Introduction

### 1.1 Cancer epidemiology

Cancer is a major threat to humanity and is described as a generic term for large group of diseases that can affect any part of the body (Siegel et al., 2019). The speed formation of abnormal cell that grow beyond their normal boundaries and which can destroy adjourning body parts and spreading to other organs is one defining feature of cancer. The later process known as metastasis is the common cause of death from cancer. Other terms used to describe the condition are neoplasm, malignant and tumor. Cancer accounted for nearly 10 million deaths in 2020 (Ferlay et al., 2021) and is an important cause to increasing life expectancy in every country of the world (Bray et al., 2021). In 2019 according to estimates from the world Health Organization (WHO) that cancer is the first or second leading cause of death before 70 years of age in 112 of 183 member countries (WHO, 2020). The overall consequences of cancer incidence and mortality is growing rapidly worldwide which reflects both aging and growth of the population as well as changes in the prevalence and distribution of the main risk factor for cancer, most of which is associated with socioeconomic development (WHO, 2020). Based on the GLOBOCAN cancer burden worldwide, 2020 estimates showed a short-term decline in cancer incidence and mortality due to many factors such as late in diagnosis and treatment of individuals due to COVID-19 pandemic that led to health system closures including screening program suspension and reduced availability and access to health care (Dinmohamed et al., 2020; Kutikov et al., 2020; Maringe et al., 2020; Moujaess et al., 2020; Corley et al., 2021). In 2020, the incidence of common cancer in terms of new cases were as follows in order of increasing cases: breast (2.26 million cases), lung (2.21 million), colon and rectum (1.93 million cases), prostrate (1.41 million cases), skin (non-melanoma) (1.20 million cases) and stomach (1.09 million cases) (de Martel et al., 2020). According to a survey, underdeveloped countries are at higher risk as 63% cancer related death were reported (de Martel et al., 2020). Cancer is a multifactorial malfunction involving genome complex modification between host and environment. The episode of cancer include independence from growth signal, uncontrolled replication, irresponsiveness to signals that halt cell division. Sustained angiogenesis, evasion of apoptosis and finally the ability to penetrate in other tissues. Most cancer deaths in 2020 were attributed to: lung (1.80 million deaths), colon and rectum (93,500 deaths), liver (830,000 death), stomach (769,000 deaths) and breast (685,000 deaths) (GBD, 2020).

### 1.2 Available cancer therapies

The objective of extraordinary research after the detection of malignancy is to find out quality method in the treatment of cancer using novel approaches. Over 60% treatment trials all going currently worldwide are centered on cancer (Wu and Chang, 2006). The choice of method of treatment and its success rate depends on the cancer type, its location and progression stage. Different procedures are involved in traditional cancer treatment process of which is divided into diagnosis and therapy of which is time consuming and consequently high cost (Chen et al., 2017; Li and Pu, 2019). Radiation based surgical knives, surgery, chemotherapy, radiotherapy are traditional methods of cancer treatments and most widely used. Modern modalities in cancer treatments include stem cell therapies, immunotherapy, anti-angiogenic, hormonal based therapy, targeted therapy, dendritic based immunotherapy and combinational regimens. The combination of diagnosis and therapy into one system known as cancer theranostic has shown great potential in the field of cancer treatment (Cheng et al., 2014; Ng and Zheng, 2015; Ali et al., 2020). The corner stone for cancer therapy is the combination of two or more chemotherapeutic treatments to specifically target cancer inducing or cells sustaining pathway (Anticancer, 2004; Yap et al., 2013). The use of monotherapy treatment is more susceptible to drug resistance because constant treatment with a single agent makes cancer cells to recruit alternative salvage pathway (Gottesman and Fojo, 2002; Khdair et al., 2010). Photo-theranostic which is optical imaging have been studied widely due to their unique advantages such as high safety and sensitivity in addition to low cost and ability of multi-channel imaging (Yin et al., 2017; Jiang and Pu, 2018; Wang et al., 2020; Zhang et al., 2020). Variety of materials such as anticancer drugs, small molecular dyes and biomacromolecules, based on photo-theranostic system have been developed (He et al., 2019; Li et al., 2020; Zhang et al., 2020).

#### 1.2.1 Chemotherapy

Use of chemotherapeutic is the important weapon of cancer and one of the most used cancer treatments that offers the best hope for cancer (Cook et al., 2016). Chemotherapeutic agent enforces apoptosis by stopping tumor progression by killing off the ability of the cancer cells to divide. The toxic effect of chemotherapy to the patient is occasioned by increasing the susceptibility to host diseases and effect on bone marrow cells, which strongly reduce immune system. In normal biological cell functioning, excess and damaged cells are refreshed through new cell formation signaling thus cell proliferation is being regulated. Chemotherapeutic agents act on tumor cells by bringing changes resulting in either death or stoppage of the growth of the cells, thus being classified as cytotoxic and cytostatic respectively. Chemotherapeutic agents also target normal cells resulting in severe adverse effects such as compromised immune system, hair loss, vomiting, nausea, weakness, complicated infections and eventually death (Rodgers et al., 2012). This is due to inhibition of growth and hair follicles cells as well as bone marrow cells and gastrointestinal tract cells by chemotherapeutic agents. There are over 132 FDA-approved chemotherapeutic agents and the use in cancer treatment started in the beginning of 20th century. Chemotherapy is mainly systemic and can be used alone or in combination therapies (Bhosle J and Hall, 2009). Due to the non-selective therapeutic approach of chemotherapeutics, it does not essentially eliminate cancer stem cells (CSCs). Because of this disadvantage, neoplasms keep a subpopulation of CSCs that give the cancer cell its self-renewal, invasive and differential potentials (Chen and Huang, 2013). Moreover, combination therapy that include agents like notch the gamma-secretase inhibitor that target CSCs would therefore attenuate the likelihood of relapse and reduce drug resistance (Takebe et al., 2015).

##### 1.2.1.1 Different types of chemotherapeutic agents

Chemotherapeutic agents can be categorized based on their chemical structures, mechanism of action and composition. Based on this category, some chemotherapeutic agents can fall into more than one group due to varied mode of action. The oncologists predict how effective a chemotherapeutic agent is by proper study on the mode of action including its side effects. Drug studies in combination chemotherapies help to decide the time, order and dosage of each drug administered in the therapy (Ewesuedo RB, 2003).

###### 1.2.1.1.1 Alkylating agents

Many cancers like lymphoma, leukemia, sarcoma, myeloma and Hodgkin’s disease are treated with alkylating agents (Of et al., 2000). The mechanism of action of alkylating agents is by direct DNA damage which stops the division of cancer cells and is efficacious in all stages of the cell cycle. On the pit fall of alkylating agents, they can cause damage to bone marrow as they damage DNA. Although rarely, long term damage can result in acute leukemia depending on the dosages used. Leukemia due to alkylating agents arises after five to 10 years of treatment. Based on the same mode of action, alkylating and platinum drugs like cisplatin, carboplatin and oxaliplatin can be grouped together but have reduced tendency to cause post treatment leukemia. Examples of alkylating agents are given in Table 1.

**TABLE 1:** Various classes of anticancer chemotherapeutic agents and their examples (Rehman, 2018).

Classes	Names of drugs
Alkylating agent	Nitrogen mustards: such as mechlorethamine (nitrogen mustard), chlorambucil, cyclophosphamide (Cytoxan^®^), ifosfamide, and melphalan
	Nitrosoureas: which include streptozocin, carmustine (BCNU), and lomustine
	Alkyl sulfonates: busulfan
	Triazines: dacarbazine (DTIC) and temozolomide (Temodar^®^)
	Ethylenimines (thiotepa and altretamine (hexamethylmelamine)
Anti-metabolites	5-Fluorouracil (5-FU), Methotrexate, pemetrexed, Pentostatin, Thioguanine
	6-Mercaptopurine (6-MP)
	Capecitabine (Xeloda^®^)
	Cladribine, Clofarabine
	Cytarabine (Ara-C^®^), Floxuridine Fludarabine, Gemcitabine (Gemzar^®^), Hydroxyurea
Anthracyclines	Daunorubicin, Doxorubicin (Adriamycin^®^)
	Epirubicin, Idarubicin
Mitotic Inhibitors	Taxanes: paclitaxel (Taxol^®^) and docetaxel (Taxotere^®^) Epothilones: ixabepilone (Ixempra^®^)
	*Vinca* alkaloids: vinblastine (Velban^®^), vincristine (Oncovin^®^), vindesine (Eldisine^®^) and vinorelbine (Navelbine^®^)
	Estramustine (Emcyt^®^)
Hormonal Chemotherapeutic Agents	Prednisone, methylprednisolone (Solu-Medrol^®^), and dexamethasone (Decadron^®^)

###### 1.2.1.1.2 Anthracyclines

Anthracyclines work by targeting DNA replication enzymes affecting cells in all cell cycle phases. They are also antibiotics. Various anticancer agents fall in the scope of this class of treatments. Exceeding a critical limit can permanently damage the heart and this is a big limitation of drugs under this group (Rehman, 2018). Examples of anthracyclines are shown in Table 1.

###### 1.2.1.1.3 Antimetabolites

Antimetabolites by incorporation, stop the growth of DNA and RNA as they are analogs for the units of DNA and RNA. This group of anticancer drugs affect the S phase of the cell in particularly and is used for the treatment of ovarian cancers, leukemia, intestinal tract and other forms of cancer (Rehman, 2018). Examples of antimetabolite agents shown in Table 1.

###### 1.2.1.1.4 Other antitumor

These are antibiotics antitumor agents that do not belong to anthracycline. Examples include actinomycin D, mitomycin C, bleomycin. This class of anticancer drugs can damage the heart at high dosage (Rehman, 2018). They can lead to post treatment acute myelogenous leukemia after two to 3 years of treatment in most cases.

###### 1.2.1.1.5 Topoisomerase inhibitors

These anticancer drugs cause the unwinding of DNA and so stop DNA replication. Most cancers like lung, leukemia, ovarian, gastrointestinal and others are treated with these drugs. Examples of topoisomerase I inhibitors are topotecan and irinotecan (CPT-II) and examples of topoisomerase II are etoposide (VP-16) and teniposide and mitoxantrone (Rehman, 2018).

###### 1.2.1.1.6 Mitotic inhibitors

These are plant alkaloids and are naturally derived products in nature. They function by inhibition of protein necessary for cell division especially in the mitotic phase of the cell cycle and subsequently damaging other phases too (Rehman, 2018). These drugs can treat the following cancers, breast, lung, lymphoma, leukemia and myelomas. Peripheral nerve damage as side effect can put limits to dosage of these drugs. Examples seen in Table 1.

###### 1.2.1.1.7 Miscellaneous chemotherapy

Some uncategorized chemotherapeutic agents belong to this group and known for uncommon modes of action. Examples include L-asparaginase, an enzyme and the proteasome inhibitor bortezom (Velcade^®^) (Of et al., 2000).

#### 1.2.2 Hormone therapy

Hormonal anticancer treatment is effective to treat cancer and is devoid of cytotoxicity which is associated with chemotherapy. Field of molecular biology advancement in recent years clarified the role of hormone therapy in cell growth and in regulation of malignant cells. About 40% of tumors in women and 25% in men are known to have hormonal basis. Hormone-like drug which are steroid are used in the treatment of cancers like lymphoma, multiple myeloma and leukemia. These steroids are also used as anti-emetic drugs to relief nausea and vomiting, they also mitigate hypersensitivity to the treatment in chemotherapy (Rehman, 2018). Examples are presented in Table 1.

#### 1.2.3 Cancer immunotherapy

Cancer immunotherapy is made up of several vital steps and has been rapidly developed as a promising strategy for cancer treatment. The so–called cancer–immunity cycle include release of cancer antigens, presentation of cancer antigens by antigen-presenting cells (APCs), priming and activation of T cells, infiltration and trafficking of T cells to tumors and finally recognition and cytotoxic T cell killing of tumor cells. Current approaches to cancer immunotherapy mainly therapeutic antibodies, adoptive cell therapy, cancer vaccines might be the potential therapeutic target with various methods (Kapadia et al., 2015).

#### 1.2.4 Radiation therapy

Radiation therapy is a distinguished field of specialization in medicine with branches such as radiation oncology (Baskar et al., 2012). This therapy can be described as a physical entity used to kill cancer cells and the kind of radiation is ionizing radiation. The energy involved in the radiation can genetically alter the cancer cells or directly kill the cancer cells so that they accede to apoptosis and cell death. In genetic alterations, the damaged DNA is unable to replicate and cell division thus halted, which in turn causes the cell to die. Radiation has adverse effect of affecting the normal cells lying in the peripheries of the main tumorous mass. However, normal cells’ ability to regain normal function faster than cancer cells minimize the net damage done by radiation through improved imaging techniques and attempts at accurate targeting of the cancer mass (Goldblum and Weiss, 2013).

#### 1.2.5 Angiogenesis inhibitors

Angiogenesis inhibitors are chemicals that can cut off blood supply to the tumor cells as nutrition to the tumor cells is provided by blood vessels. Examples of this agent are interferon, bevacizumab, thalidomide and cediranib, these drugs can be administered sometimes in combination with chemotherapeutic drugs to increase therapeutic outcome (Shojaei, 2012).

### 1.3 Limitations of available cancer therapies

Although all the available cancer therapies described in Section 1.2 have been proven to be efficacious in various degrees in cancer therapy, they all have limitations. In Section 2.2, the limitations of chemotherapy, hormone therapy, cancer immunotherapy, anti-angiogenesis inhibitors and radiation therapies in cancer treatment are discussed alongside the application of nanogels to overcome those limitations.

## 2 Nanogels for cancer therapy

In recent years, nanogels have been proposed as potential drug delivery systems to overcome limitations of available conventional cancer therapies. The properties of nanogels and their applications as targeted drug delivery systems for cancer therapy will be discussed in Section 2.1.

### 2.1 Properties of nanogels

According to Soni et al. (2016), “nanogels are three-dimensional hydrogel materials in the nanoscale size range formed by cross-linked swell-able polymer networks with a high capacity to hold water, without actually dissolving into the aqueous medium.” Nanogels possess the combined characteristics of nanoparticles and hydrogels, and their size ranges from 20 to 200 nm (Jain et al., 2019; Abdolahinia et al., 2022). The gel feature of nanogels enables them swell when in contact with physiological fluids. This makes them flexible enough to be in close proximity with the targeted site and enhance diffusion of drugs and other therapeutics. In addition, the nanogels can be formulated for drug release under specific physiological conditions (Mauri et al., 2021). As nanoparticles, they possess the ability of crossing biological barriers and membranes with ease. They have been proven to be instrumental in the administration of drugs across the blood-brain barrier (Peng et al., 2021). They also have the ability to evade the reticuloendothelial system (Sultana et al., 2013). Nanogels are therefore characterized by high stability, biodegradability and biocompatibility (Lai and He, 2016). The several unique characteristics of nanogels makes them versatile and suitable to be developed into various dosage forms for the potential treatment of several diseases including, but not limited to, cardiovascular diseases, cancers and other malignancies, to mention a few (Prajnamitra et al., 2019; Howaili et al., 2021a). Nanogels can also be applied in medical imaging and theranostics (Peng et al., 2019).

### 2.2 Applications of nanogels as improved drug delivery systems for cancer therapy

Nanogels have been proven as one of the excellent DDS with the ability to overcome the short-falls of conventional anti-cancer therapies and improve cancer treatment outcomes. For instance, as described in Section 1.2.1, one of the major short-falls of conventional chemotherapy is its non-selective mechanism which targets both cancerous and non-cancerous cells. This leads to increased adverse effects and toxicity. Stimuli-sensitive nanogels have been successfully developed and evaluated for targeted delivery of chemotherapeutic drugs to cancer cells with minimal adverse effects and reduced toxicity (Lv et al., 2018). Hormone therapies are efficacious for cancers associated with hormones however, some hormone therapies have been associated with increased risk factors for diabetes mellitus (Ye et al., 2021) and blood clots (Heit et al., 2002). Such risk factors are not associated with nanogels formulated for targeted drug delivery in such cancer types. Loaded nanogels of chitin-polymerized doxorubicin have been successfully utilized in the management of breast, liver, prostrate and lung cancer (Kaur et al., 2014). Cancer immuno-therapy is an improvement over many conventional cancer therapies, however, there are several physical barriers and metabolic factors limiting optimal cancer immune-therapy (DePeaux and Delgoffe, 2021) which are not applicable in the use of nanogels. In addition, cancer immuno-therapy could increase toxicity to non-cancerous cells. Tang et al. (2018) succeeded in developing protein nanogels to deliver optimum quantities of chimeric antigen receptor (CAR) T-cells for immunotherapy. The nanogels were designed to respond to T-cell receptor (TCR) activation by releasing optimum quantities of CAR T-cells into the tumor microenvironment. The release of proteins was modulated to ensure the significant release of drug cargo which increased efficacy without increasing toxicity. Their research results reveal the potential use of nanogels in T-cell immunotherapy. Angiogenesis inhibitors are efficient to some extent in arresting the growth of certain which require production of new blood vessels. However, they may not be efficient if the cancer utilizes existing blood supply (Saman et al., 2020). The efficient tumor-targeting ability of nanogels overcomes this limitation of angiogenesis inhibitors. Su et al. (2013) synthesized thermo- and pH-responsive poly (N- isopropyl acrylamide co-acrylic acid) nanogel for tumor targeting. Radiation therapy is also shares the same limitation with most conventional cancer therapies which is non-selectivity and subsequent cell toxicity (Wang and Tepper, 2021). In addition to reducing cell toxicity by ensuring targeted drug delivery, nanogels can be formulated to combine the benefits of two or more conventional cancer therapies. The novel research by Liu et al. (2018) reported a biodegradable and pH-sensitive nanogel system as a drug nano-carrier for combinational chemotherapy and radiotherapy. This stable and uniform nanogel was fabricated through self-assembly of carboxymethyl cellulose and bovine serum albumin. The successful loading of the radiounuclide, ^131^I and camptothecin into a hybrid nanogel showed a high drug loading pH-controlled drug release profile capacity of 16.72 wt% with excellent biocompatibility and low hemolysis. This formulation enhanced drug accumulation at the tumor site, improved cell uptake and prolonged circulation in the blood. Tyler et al. (2010) proved that OncoGel containing 6.3 mg/ml paclitaxel in 18 Fischer-344 rats bearing glioma was safe for intracranial injection but most effective when administered with radiation therapy. pH and temperature-sensitive poly (N- isopropyl acrylamide co-acrylic acid) carrying citric acid and coated Fe_3_O_4_ nanoparticles were conjugated with Cy 5.5-labelled lactoferrin which served as bi-functional contrast agent for both MRI and intraoperative optical imaging for glioma (Jiang et al., 2013). A thermo-sensitive hydrogel co-loaded DOX/IL-2/IFN-γ has been reported, which showed improved therapeutic efficacy of B16F10 melanoma tumor by increasing tumor cell apoptosis and increasing proliferation of the CD3+/CD4+T cell and CD3+/CD8+Tcells (Lv et al., 2018).

### 2.3 Classification of nanogels

**FIGURE 1:**
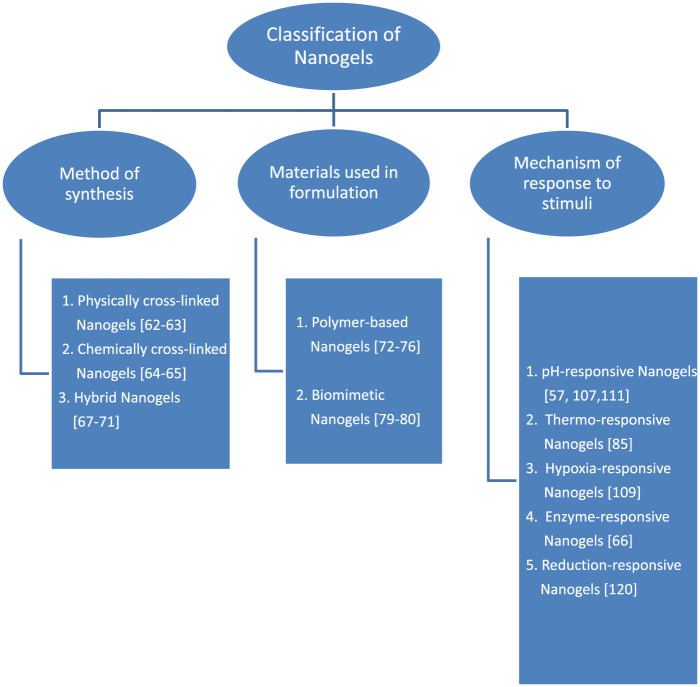
Classification of nanogels.

Nanogels can be classified based on the method of synthesis, materials used in their formulation and the mechanism of nanogel response to stimuli. Figure 1 is a schematic diagram on classification of nanogels.

#### 2.3.1 Nanogel classification based on method of synthesis

**FIGURE 2:**
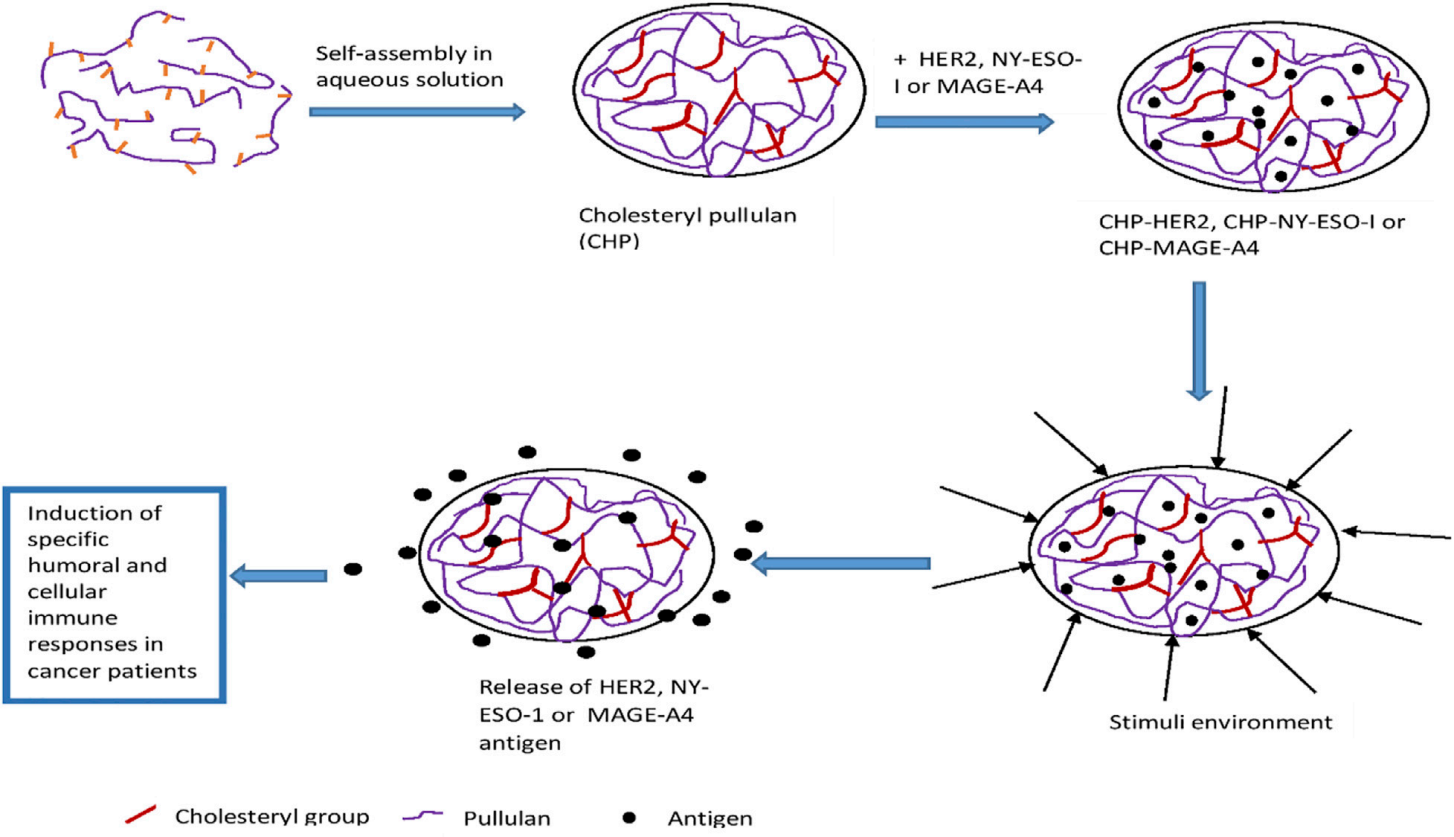
Preparation and mechanism of antigen release from CHP nanogel based vaccine.

Nanogels are made up of several networks consisting of linked matrices which enable them to retain their structures and ensure optimal drug release when exposed to physiological fluids. They can be classified into either physically or chemically cross-linked nanogels based on their method of synthesis (Theune et al., 2019; Yin et al., 2020) (Figure 2).

##### 2.3.1.1 Physically cross-linked nanogels

Physically cross-linked nanogels are supramolecular particles consisting of polymer molecules formed through non-covalent interactions (Yin et al., 2020). Physical cross-linking is usually through ionic, hydrophilic-hydrophobic balance, Van der Waals and hydrogen bonds. They do not require crosslinking agents. This is an advantage because most crosslinking agents are associated with unwanted interactions during nanogel formation and the encapsulation of bioactive materials. Crosslinking agents can also adversely affect the performance of drug loading in nanogels and have been associated with toxic effects. In addition, the size of nanogels can be customized for efficient drug delivery by modification of physical conditions during formulation (Mauri et al., 2021). Theune et al. (2019) synthesized nanogels *via* physical-crosslinking using polymer Polypyrrole (PPY) which was “incorporated into dendritic polyglycerol (dPG) cross-linked poly (*N*-isopropylacrylamide-*co*-*N*-isopropylmethacrylamide) nanogels (p (NIPAm-*co*-NIPMAm)) by semi-interpenetration to form the PPY/Co-dPG nanogels.”

##### 2.3.1.2 Chemically cross-linked nanogels

Chemically cross-linked nanogels consist of polymers formed through covalent chemical interactions (Chouhan et al., 2020). The starting materials are low-molecular-weight monomers, polymer precursors or polymers with specific terminal or pendular reactive groups (Mauri et al., 2021). The linkages formed by chemical cross-linking methods are more stable than those formed by physical methods as a result of the stronger, irreversible bonds formed through covalent interactions (Yin et al., 2020). Chemical crosslinking methods include “polymerization by emulsion, reversible addition-fragmentation chain transfer (RAFT), click chemistry crosslinking, and photo-induced crosslinking” (Kabanov and Vinogradov, 2009). Yang et al. (2021) developed “enzyme-responsive photo-cross-linked nanogels (EPNGs) through UV-triggered chemical cross-linking of cinnamyloxy groups in the side chain of PEGylation hyaluronic acid (HA) for CD44-targeted transport of cytochrome *c* (CC).” The nanogels were efficacious against cancer cells with minimal toxicity to non-cancerous cells.

##### 2.3.1.3 Hybrid nanogels

Hybrid nanogels are defined as “composites of nanogel particles dispersed in organic or inorganic matrices” (Wu et al., 2014). Composites are materials made up of two or more components intimately merged together to combine the advantages of each individual component. For instance, a hydrophilic component can be combined with a hydrophobic component to enable the loading of both hydrophilic and hydrophobic drugs into the hybrid nanogel. Likewise, easily degradable drugs can be protected by incorporating them into an inner core made of suitable materials and polymers while an outer shell could be fabricated with different materials to protect the drug in the inner core until it arrives the targeted drug delivery sites. Some stimuli-responsive polymers can also be incorporated into hybrid nanogels to facilitate targeted drug delivery. The major concept of hybrid nanogels is the strategic combination of polymers and other materials to produce multifunctional nanogels (Thoniyot et al., 2015; Ma et al., 2017). Morimoto et al. (2005), synthesized hybrid nanogels by polymerizing cholesteryl group-bearing pullulan (CHP) with methacryloyl groups (CHPMA) nanogel with 2-methacryloyloxyethyl phosphorylcholine (MPC). The nanogels had dual cross-linking structure that featured physical and chemical cross-linking processes with the cholesteryl groups and the MPC polymer chains respectively (Table 2). The “nanogels maintained the ability of trapping and releasing enzymes by host-guest interaction of cholesteryl group and cyclodextrin.” Wu et al. (2010) developed core−shell structured hybrid nanogels composed of a Ag nanoparticle (NP) as core and smart gel of poly (*N*-isopropylacrylamide-*co*-acrylic acid) as shell to ensure optimal drug concentrations at tumour sites and cancer cell imaging. The nanogels were designed to shrink in respond to pH stimulus to enable increase in UV-vis absorption intensity for cancer cell imaging. The physicochemical properties of the inner core and outer shell enabled the nanogel penetrate into intracellular spaces for potential targeted and optimal anti-cancer drug delivery.

**TABLE 2:** Current clinical trials on Nanogel based cancer vaccine candidates.

Nanogel Based Vaccine Candidate	Adjuvant/Additive	Cancer Type	Clinical Phase	Reference
CHP-HER2		Lung, breast, pancreatic, nasal cavity, pelvis cancers	I	Kitano et al. (2006)
	GM-CSF/OK-432	Breast, ovarian, non-small cell lung cancer	1	Kageyama et al. (2008)
CHP-NY-ESO-1		Esophageal, prostate, malignant melanoma	1	Kawabata et al. (2007), Tsuji et al. (2008), Uenaka et al. (2007), Wada et al. (2008)
		Esophageal cancer	1	Kageyama et al. (2013), Kawada et al. (2012)
	OK-432	Advanced esophageal cancer	I	Aoki et al. (2009)
	MIS416, anti-PD-I	Urothelial, prostate, synovial sarcoma, malignant solid tumors	I	Ishihara et al. (2020)
	Poly ICLC, Anti-PD-1	Esophageal	1	Ishikawa et al. (2021)
CHP-NY-ESO-1 plus CHP-HER2	OK-432	Esophageal cancer	1	Aoki et al. (2009)
CHP-MAGE-A4	OK-432	Esophageal, lung and gastric cancers	1	Saito et al. (2014)
	OK-432	Colon, esophageal, papilla of Vater, breast, pancreatic cancers	I &II	Kyogoku et al. (2016)
	OK-432	Colon, rectal, breast, bile duct, gall bladder, pancreatic or mesothelioma	I & II	Kengo Miyauchi1 et al. (2016)
	OK-432	Colorectal, breast, bile duct, gall bladder, pancreatic or mesothelioma	I &II	Wada et al. (2018)
	OK-432	Esophageal carcinoma, head/neck, ovarian, duodenal cancers	I	Ueda et al. (2018)

#### 2.3.2 Nanogel classification based on formulation materials

Nanogels are formulated with a varied range of materials. In drug delivery, the materials used in nanogel formulations are selected based on their safety profile, compatibility with the active pharmaceutical ingredients and their potential application. These materials can also affect the characteristics and stability of the finished nanogel formulation (Sultan et al., 2021).

##### 2.3.2.1 Polymer-based nanogels

Nanogels are formulated with natural, or synthetic polymers or a combination of natural and synthetic polymers (Sultan et al., 2021). Natural polymers are obtained from nature. Some examples of natural polymers used in nanogels include cellulose, chitosan, gelatin, pullulan, and hyaluronic acid. Natural polymers have desirable characteristics of being biodegradable, non-toxic, biocompatible and abundant in nature (Kang et al., 2019; Moraes et al., 2021; Pan et al., 2021; Radwan and Ali, 2021). Synthetic polymers are (Sung and Kim, 2020) usually by-products from petro-chemical processes. However, unlike natural polymers, they are not easily renewable. There have been concerns about their limited biocompatibility with physiological with physiological membranes and their non-degradability and possible cytotoxicity (Sultan et al., 2021). Examples of synthetic polymers include poly (N-isopropylacrylamide), poly (N-isopropylacrylamide-co-acrylic acid), poly (ethylene glycol)-b-poly (methacrylic acid), poly (ethylene glycol)-co-Methacrylamide-co-Acrylic acid, and poly (2-(*N*, *N*-dimethylamino) ethyl methacrylate). Table 3 provides examples of some polymers used in nanogel formulation, their sources, and characteristics.

**TABLE 3:** Examples of some polymers used in nanogel formulations.

Polymers	Sources	Characteristics	Applications
Cellulose	Marine animals (e.g., tunicates) and plants (e.g., wood, cotton, wheat straw) (Thoniyot et al., 2015)	Renewability, biodegradability and environmental friendliness [ 68]	Cellulose-based nanogels for controlled release of doxorubicin hydrochloride (Sultan et al., 2021)
Chitosan (CS)	“Hydrolyzation of the amino-acetyl groups of chitin, obtained from crustaceans and insects, by an alkaline treatment” [ 74]	Biocompatibility, biodegradability, nontoxicity, and pH sensitivity (Kang et al., 2019; Moraes et al., 2021; Pan et al., 2021)	Rutin-loaded Chitosan/poly (acrylic acid) nanogel by gamma radiation-induced polymerization (Ma et al., 2017)
Gelatin	Partial hydrolysis of collagen from natural sources like animal bones (Morimoto et al., 2005)	Biocompatibility, biodegradability, low antigenicity, and multi-functionality (Gao et al., 2016; Liwei et al., 2019; Sung and Kim, 2020; Raza et al., 2021)	Doxorubicin-loaded nanogels using fish gelatin methacryloyl (Morimoto et al., 2005)
Pullulan	“Derived from fermentation of black yeast like *Aureobasidium pullulans*” (Nagel et al., 2020)	Non-toxicity, immunogenic, good biocompatibility and biodegradability (Nagel et al., 2020)	Pullulan-based nanogel complex was formed for efficient mi RNA delivery (Wu et al., 2010)
Hyaluronic acid	“Non-sulfated glycosaminoglycan found throughout the connective, epithelial, and neural tissues”(Qiao et al., 2019; Cao et al., 2021)	Biocompatibility, biodegradability, non-immunogenic, non-inflammatory, and non-toxic (Bang et al., 2019; Qiao et al., 2019)	Hyaluronic acid-based nanogel was formed for successful delivery of curcumin and simvastatin (Rancan et al., 2019)
Carbopol^®^ 971P NF	“Polymers of acrylic acid cross-linked with allyl sucrose or allyl pentaerythritol” (Manzanares-Guevara et al., 2020)	Low toxicity, minimal irritation, bio-adhesive, extended-release modifier, rheology modifier, stabilizer (Qu et al., 2019; Manzanares-Guevara et al., 2020)	Carbopol^®^ 971P NF-based nanogel was formed for successful delivery of low-dose, prolonged-release trans-dermal administration of Artemether (Nnamani et al., 2021)
Poloxamer 407	“Polymers of acrylic acid cross-linked with allyl sucrose or allyl pentaerythritol” (Manzanares-Guevara et al., 2020)	Low toxicity, minimal irritation, bio-adhesive, extended-release modifier, rheology modifier, stabilizer (Qu et al., 2019; Manzanares-Guevara et al., 2020)	Carbopol^®^ 971P NF-based nanogel was formed for successful delivery of low-dose, prolonged-release trans-dermal administration of Artemether (Nnamani et al., 2021)
Prosopis Africana peel powders (PAPPs)	“Polymers of acrylic acid cross-linked with allyl sucrose or allyl pentaerythritol” (Manzanares-Guevara et al., 2020)	Low toxicity, renewable, minimal irritation, bio-adhesive, extended-release modifier, rheology modifier, stabilizer (Qu et al., 2019; Manzanares-Guevara et al., 2020)	Carbopol^®^ 971P NF-based nanogel was formed for successful delivery of low-dose, prolonged-release trans-dermal administration of Artemether (Nnamani et al., 2021)
Poly (N-isopropyl acrylamide) (PNIPAm)	*N*-isopropylacrylamide chemical compound (Cho et al., 2019)	Smart, thermo-responsive, flexible physical properties, possible toxicity (Cho et al., 2019; Ye et al., 2019; Massi et al., 2020)	Poly (N-isopropylacrylamide) Nanogels for efficient tumor therapy (Massi et al., 2020)
Poly (N-vinylcaprolactam) (PVCL)	N-Vinylcaprolactam (Ye et al., 2019; Sawada et al., 2020)	Smart, thermo-responsive, biocompatible (Ma et al., 2019)	PVCL-based nanogels for potential HIV therapy (Sawada et al., 2020)
Poly (2- (*N*, *N*-dimethylamino) ethyl methacrylate) (PDMAEMA)	Dimethylamino-ethyl methacrylate chemical compound	Smart, pH-responsive, versatile applications (Tran et al., 2018)	PDMAEMA nanogels for targeted delivery of Doxorubicin in cancer therapy (Liu et al., 2022)
Poly (lactic-co-glycolic acid) (PLGA)	Lactic-co-glycolic acid chemical compound	Biodegradable, biocompatible, biosafety, thermoplastic (Howaili et al., 2021b)	PLGA hydrogels for controlled delivery of Temozolomide in cancer therapy (Shang et al., 2022)

##### 2.3.2.2 Biomimetic nanogels

Biomimetic nanogels have their surfaces coated with cellular membranes and other biological cellular components to mimic them. This enables them to evade elimination by the reticuloendothelial system and makes it possible for biomimetic nanogels to pass through physiological barriers for targeted drug delivery. They have been proven to exhibit high specificity and potency in cancer therapy (Raza et al., 2021). They are also reported to have a very good safety profile. This makes them invaluable in nanogel formulations. Biomimetic nanogels can be developed with cell membranes of platelets, and mesenchymal stem cells (Gao et al., 2016; Liwei et al., 2019). Gao et al. (2016) reported the efficacy of their stem cell membrane-coated nanogels in delivery of doxorubicin when compared with the free drug.

#### 2.3.3 Nanogels classification based on response to stimuli

Nanogels can be classified as stimuli-responsive nanogels based on their mechanism of drug release. Nanogels can be formulation to respond to different physical and environmental stimuli when ingested into the body. This feature of nanogels makes is possible for controlled and targeted drug delivery. Nanogels could be designed to release drugs only under certain physical or physiological conditions. These conditions may include specific temperatures, pH, magnetic fields, ultra-violet light, reduction reaction, oxidation-reaction, hypoxia conditions, among others (Sultan et al., 2021). Stimulus response may initiate the interactions within the nanogel framework or cleavage of physical or chemical bonds formed during the design of the nanogel, and swelling of the nanogel matrix which subsequently leads to drug release. It may also lead to degradation of the nanogel materials in which the drug is encapsulated, leading to drug release. The ability of the nanogels to respond to specific stimuli for instance, hypoxia-responsive stimuli, enables targeted drug delivery to tumor sites which are usually located in hypoxic micro-environment (Peng et al., 2019; Peng et al., 2021). Stimulus-responsive nanogels can also be formulated with polymers that interact with cells at the target sites to form imaging for diagnosis and monitoring of cancer treatment (Soni et al., 2016). Some nanogels can be developed to respond to one stimulus (single-responsive stimulus), two stimuli (dual-responsive stimuli), or three stimuli (triple-responsive stimulus) (Nagel et al., 2020; Cao et al., 2021; Peng et al., 2021).

##### 2.3.3.1 Degradable stimuli-responsive nanogels

Degradable stimuli-responsive nanogels are designed by cross-linking bonds that can disintegrate within the nanogel framework. When exposed to specific stimuli, the nanogel frame degrades through different mechanisms for targeted drug delivery. Qiao et al. (2019), prepared folated pH-degradable nanogels containing docetaxel (an anti-cancer drug) and indoleamine 2,3-dioxygenase 1 inhibitor NLG919 for simultaneous chemo-immunotherapy. They modified poly (vinyl alcohol) (PVA) with vinyl ether acrylate (VEA) groups for UV- cross-linking and acidic degradation, and introduced carboxyl groups through alteration of succinic anhydride to improve drug loading. They also used folic acid ligands to facilitate tumor targeting. The pH in the cancer micro-environment triggered significant release of the therapeutic agents leading to tumor cell deaths and a synergistic release of CD8^+^ T cells and natural killer (NK) cells for immunotherapy when compared to the free drug controls.

### 2.4 Applications of nanogels in drug delivery and therapeutics

#### 2.4.1 Enhancement of drug solubility

Nanogels have been used to successfully enhance the solubility of poorly soluble drugs, thereby improving their bioavailability. Bang et al. (2019) reported increased solubility of poorly water-soluble anti-cancer drug, Curcumin when fabricated into nanogels. They developed a nano-matrix with hydrophobic and hydrophilic components which ultimately increased Curcumin solubility in water by 20,000 times, hence, improving its bioavailability.

#### 2.4.2 Improved absorption of drugs with high molecular weights

Nanogels have been reported to be effective in improving absorption of drugs with high molecular weights. Tacrolimus, is an immune-suppressive drug with high molecular weight. Rancan et al. (2019) developed polyglycerol-based thermo-responsive tacrolimus nanogels for dermal application. The tacrolimus was incorporated into an aqueous suspension of the nanogels and applied on excised skin for evaluation of anti-proliferative effects. The thermo-responsive characteristics of the nanogel enhanced adsorption of tacrolimus through the skin due to the slight increase in the body temperature associated with inflammation. The results revealed the nanogel formulation of tacrolimus exhibited significant anti-proliferative effects.

#### 2.4.3 Targeted drug delivery

The nano-particulate dimension of nanogels provides them with unlimited prospects for targeted drug delivery. Targeting the delivery of drugs reduce drug losses within physiological spaces and enhances optimal drug concentration at targeted sites. In addition, targeted drug delivery reduces adverse drug reactions which may lead to poor adherence to medications. Manzanares-Guevara et al. (2020) developed nanogels using for targeted delivery of curcumin in colon cancer. They combined different materials using several methods to achieve targeted drug delivery with promising future applications.

#### 2.4.4 Controlled drug delivery

The vast range of materials used in nanogel formulations and their nano-sizes of nanogels produce versatile products which can be modified for controlled drug delivery. Qu et al. (2019) described the synthesis of a promising nanogel for “on-demand” and controlled delivery of camptothecin to tumor sites.

#### 2.4.5 Possibilities of new dosage forms of existing drugs

Nanogels have also provided opportunities for exploring new dosage forms of existing drugs. Artemether is currently available in oral and injectable dosage forms. Nnamani et al. (2021) successfully formulated and characterized nanogels for trans-dermal administration of lower-dose artemether. The formulated nanogel proved that artemether can be administered through the skin. The formulated nanogel had the potential to eliminate the side effects associated with oral and injectable dosage forms of artemether. Furthermore, a prolonged drug release was also achieved to prevent frequent drug administration associated with available marketed formulations of artemether. Furthermore, the versatile nature of nanogels enabled Cho et al. (2019) to develop 3-dimensional (3 D)-printed nanogel discs containing paclitaxel and rapamycin for adjuvant ovarian cancer therapy.

#### 2.4.6 Improved protein and gene delivery

Nanogels have also been reported to be efficient in the administration of proteins for therapeutic purposes. Massi et al. (2020) developed nanogels for protein delivery. The nanogels were stimulated to release the peptides in response to matrix metalloproteinases (MMP)-7 for enzyme–specific protein delivery. Ye et al. (2019) also developed nanogels with promising potential for transfection of DNA. Sawada et al. (2020) also reported the formulation of a nanogel hybrid assembly for exosome intracellular delivery. They developed an amphiphilic cationic nanogel which was mixed with exosomes from mouse macrophage cells to form a hybrid. The entire process was traced to confirm the successful delivery of the exosomes into cells.

#### 2.4.7 Improved activation of pro-drugs

When compared with other nanoparticles, nanogels have been useful in reducing the activation time of pro-drugs. Gemcitabine requires activation to be efficacious in cancer therapy. Ma et al. (2019) synthesized a DNA-like polygemcitabine (**Ge**_10_) nanogel, which rapidly converted Gemcitabine to its active derivative.

#### 2.4.8 Drug combination therapies

Nanogels have also been reported to be efficient in drug combination therapies. This feature is particularly important in reducing pill burdens, improving treatment outcomes of different disease conditions. Tran et al. (2018) developed nanogel formulations containing paclitaxel and 5-fluorouracil using poly (ethylene glycol) methyl ether (mPEG) and chitosan for combination cancer therapy. Although great progress has been made in improving the efficacy of cancer treatment through combination treatment using drug agents, there are still challenges in improving the efficiency of drug delivery. Liu et al. (2022) reported a study in which olaparib and doxorubicin were co-loaded on disulfide bond cross-linked polypeptide nanogels for the treatment of breast cancer in mouse models. Under stimulation of a high glutathione environment in cancer cells, the drug is quickly released from the nanogel to target cancer cells. In addition, compared with free drugs and single-drug-loaded nanogels, dual-drug- co-loaded nanogels exhibit the best anti-cancer effect and demonstrated excellent biological safety. Therefore, the co-delivery of olaparib and doxorubicin through polypeptide nanogels presents good prospects for application as anti-cancer treatment.

#### 2.4.9 Advancement in simultaneous combination of chemotherapy and other anti-cancer therapies

Emerging formulation technologies have led to advancement in simultaneous combination of chemotherapy and other anti-cancer therapies through nanogels. Howaili et al. (2021b) developed nanogel for dual delivery of chemotherapeutic curcumin and photothermal therapy for cancer treatment. Shang et al. (2022) developed a nanogel to “co-deliver chemotherapeutic paclitaxel (PTX) and immunotherapeutic agent interleukin-2 (IL-2) under mild conditions for combinational treatment of triple-negative breast cancer.”

#### 2.4.10 Potential reversal of drug resistance

Drug resistance in cancer therapy is a major limitation to efficient drug delivery to cancer cells and improved clinical outcomes. Ma et al. (2021) reported the development of hyaluronate (HA) nanogels containing doxorubicin and cisplatin to reverse drug resistance. The nanogel was designed to maintain optimal drug concentrations within the tumour cells. The evaluation of the nanogels revealed the potential of the nanogels in reversing drug resistance.

### 2.5 Applications of nanogels in targeted cancer therapy

As discussed in Section 1, cancer is a non-communicable disease with one of the highest morbidity and mortality rates globally (World Cancer Research Fund/American Institute for Cancer Research, 2018; Wild et al., 2020). Most of the current cancer therapies have been reported to have limitations including: poor bioavailability, slow activation of pro-drugs, unpredictable drug release mechanisms. In addition, many of them have numerous adverse drug reactions which decrease patients’ medication adherence and subsequently lead to poor cancer treatment outcomes (Tran et al., 2018; Ma et al., 2019). In order to meet the United Nation’s sustainable development goals which includes “reducing by one-third, premature mortality from non-communicable diseases including cancer by 2030” (United Nations Sustainable Development Goals, 2021), innovative cancer therapies must be prioritized. One of such innovative cancer therapies is the emerging potential application of nanogels in targeted cancer therapy. Nanogel formulations of anti-cancer drugs have extended the horizon for what might be one of the most important revolutions in targeted cancer therapies.

#### 2.5.1 Nanogels formulations for lung cancer therapy

Lung cancer is one of the most diagnosed type of cancer. Tobacco smoking has been reported to be the major causative factor for the high incidence of lung cancer globally (Wild et al., 2020). Literature searches revealed few proposed nanogels formulations for lung cancer therapy.

##### 2.5.1.1 Multi-functional nanogels for lung cancer therapy

Cisplatin (chemical name: cis-diamminedichloroplatinum (II), is an efficacious anti-cancer drug (Cancer Drug Resistance Research Perspectives Torres, 2013; Aldossary, 2019). Cisplatin is the first-line treatment for different types of solid tumors, including lung cancers. However, cisplatin resistance poses a challenge in cancer therapy because it also results in poor treatment outcomes due to insufficient drug activity and apoptosis induction (Giacomini et al., 2020; Sun et al., 2020). Sun et al. (2020) developed a multi-functional Valproate-D-nanogel to enhance apoptosis induction of cisplatin. The nanogel successfully reversed cisplatin-resistance in human lung adenocarcinoma cancer, with a resistance reversion index (50.22). They concluded that the nanogel could effectively inhibit cisplatin-resistance.

##### 2.5.1.2 pH-responsive nanogels for lung cancer therapy

Ginsenoside Compound K (CK) is a secondary ginsenoside bio-transformed from major ginsenosides and serves as a potential anti-cancer compound (Sharma and Lee, 2020). However, it is poorly water-soluble with low bioavailability. Hence, it has limited applications in cancer therapy. Xue et al. (2021) developed pH–responsive nanogels containing Ginsenoside Compound K for potential use in lung cancer treatment. The optimum drug release profile was determined by conducting drug release studies under a range of pH conditions. The overall anti-tumor efficacy of the formulated nanogels was higher than that of free drug by 7.7%. This confirms that the bioavailability of CK can be improved when formulated as nanogels.

#### 2.5.2 Nanogels formulations for breast cancer therapy

Breast cancer is the most diagnosed type of cancer in women. Certain physiological conditions have been reported to increase susceptibility to breast cancer (Wild et al., 2020). A few nanogel formulations have been proposed for breast cancer therapy.

##### 2.5.2.1 Hypoxia-responsive nanogel of enzymes for breast cancer therapy

Hypoxia is one of the hallmarks of cancer micro-environments. It also reduces the efficacy of chemotherapeutic agents. Developing hypoxia-responsive nanogels is an innovative strategy to turn an apparent disadvantage into an advantage. The nanogels are designed to respond only to hypoxia in cancer micro-environments aiding the efficacy of targeted drug delivery (Jing et al., 2019). A few anti-cancer agents have been developed into hypoxia-responsive nanogel formulations. One of such anti-cancer agents, Ribonuclease A (RNase) has been reported to possess high efficacy and specificity but is unstable, has a short half-life and poor membrane penetration (Si et al., 2020). Si et al. (2020) developed a hypoxia-responsive Ribonuclease A (RNase) nanogel to improve its stability, and membrane penetration. They prepared the nanogels using host-guest interactions between azobenzene (Azo) and β-cyclodextrin (βCD) conjugated to poly (L-glutamic acid)-graft-poly (ethylene glycol) methyl ether (PLG-g-mPEG). The RNase was loaded inside the nanogels in mild aqueous conditions.

The nanogel released 75% of the RNase under hypoxic conditions within the cancer micro-environment. The tumor suppression rate (TSR%) of the free RNase was 0% while that of the RNase nanogel was 68.7%. They were able to achieve a higher TSR% of 91.7% for the RNase nanogel by incorporating vascular disrupting agents into the nanogel. This confirms that a significant increase in TSR% can be achieved when unstable anti-cancer drugs with poor membrane penetration are formulated as hypoxia-responsive nanogels.

##### 2.5.2.2 Dual-targeted Nanogels for protein therapy in breast cancer

Chen et al. (2019) developed “epidermal growth factor receptor (EGFR) and CD44 dual-targeted hyaluronic acid nanogels (EGFR/CD44-NGs) that enhanced targeted delivery of protein therapy for metastatic 4T1 breast cancer *in vivo*.” Analysis of the evaluation revealed there was over 6-fold higher cellular uptake of the protein from the dual-targeted nanogels when compared with the mono-targeted nanogels. The results concluded that dual-targeted protein therapy an efficacious therapy for breast cancer metastasis.

#### 2.5.3 Nanogel formulations for skin cancer therapy

Cancers of the skin are the most common cancer type in humans. The term “skin cancer” “covers a range of pathological entities that arise from different cells of the *epidermis* and dermis” (Wild et al., 2020).

##### 2.5.3.1 Biocompatible nanogels for topical chemotherapy of aggressive melanoma

Melanoma, is the most aggressive type of skin cancer (Wild et al., 2020). Low selectivity, poor efficacy and the physiological nature of the skin constitute hindrances to topical application of chemotherapeutic agents (Sahu et al., 2019). Sahu et al. (2019) developed a chitosan-based pH-responsive biodegradable nanogel (FCNGL), encapsulated with 5-fluorouracil (5-FU) against melanoma. The nanogels were designed to release drugs in the slightly acidic cancer microenvironment leading to targeted drug release at the tumor site. The nanogels also maintained the integrity of the skin layer when compared with other conventional melanoma formulations. In summary, the study confirms that the potential of topical 5-FU nanogels in improving efficacy through targeted drug delivery for melanoma.

#### 2.5.4 Nanogel formulations for colorectal cancer therapy

Colorectal cancer is the third most diagnosed cancer type globally. Although the mortality rate has reduced, there are still concerns that lifestyle factors may increase its rate in some regions (Wild et al., 2020).

##### 2.5.4.1 Biomimetic nanogels for colorectal cancer

Biomimetic nanogels have been described in Section 2.3.2.2. Irinotecan is a camptothecin derivative targeting topoisomerase 1. Although Irinotecan was first approved for cancer therapy in Japan 25 years ago, it is still very efficacious and relevant in the treatment of metastatic and solid tumors including colorectal cancer (Bailly, 2019). Liwei et al. (2019) developed platelets membrane-camouflaged irinotecan-loaded gelatin nanogels for *in vivo* colorectal carcinoma therapy. The irinotecan (IRN)-loaded gelatin nanogels (GN) were formulated as inner cores while the outer cores consisted of platelets membranes (PTM). Since the outer core comprised of a natural membrane, the entire complex PTM/GN/IRN effectively evaded clearance by the reticuloendothelial system. The platelet also possessed the ability to accumulate at tumor sites, hence, it ensured accumulation of the complex at the tumor sites. They were able to prove that nanogels had the potential of improving drug delivery to solid tumors like colorectal cancer.

##### 2.5.4.2 Dual stimuli-responsive nanogels for colon cancer combination therapy

Dual stimuli-responsive nanogels have been described in Section 2.3.3. As described in Section 2.4.8, nanogels have great potential in combination therapies. Abedi et al. (2021) fabricated smart dual-responsive nanogels for efficient and controlled release of doxorubicin (DOX) and curcumin (CUR) in HT-29 colon cancer cells. The DOX/CUR-hydrogels or DOX/CUR-nanogels were prepared using two different loading methods of DOX and CUR into the P(NIPAAm-co-DMAEMA). In the first method, the fabrication of DOX/CUR-HGs was conducted by adding DOX-HCl to well-dispersed hydrogel. The mixture was centrifuged. Surfactants were added to the dissolution medium to improve the water-solubility of CUR before incorporating it into the mixture. The second method included the fabrication of DOX and CUR-loaded nanogels (DOX/CUR-NGs) *via* a modified water-in-oil-in-water (W/O/W) emulsion technique. In both methods, the DOX/CUR feeding ratio 1:1 was used. The nanogels were pH and thermo-responsive. The results of the experiment showed that the nanogel exhibited more anti-tumor efficacy than the individual drug formulations. Hence, simultaneous delivery of the dual drugs through the pH and thermos-responsive nanogels could synergistically potentiate the anti-tumor effects on colon cancer.

#### 2.5.5 Nanogel formulations for prostate cancer therapy

Prostate cancer is the second most common cancer type in men globally. The morality rate is declining due to early diagnosis and treatment (Wild et al., 2020).

##### 2.5.5.1 DNA aptamer-modified nanogel for targeted prostate cancer therapy

Aptamers are short single-stranded DNA or RNA oligonucleotides that can selectively bind to small molecular ligands or protein targets with high affinity and specificity (Kaur et al., 2018). Aptamers (Apt) can efficiently deliver proteins, drugs or nucleic acids into specific structures in cells by conjugating to small interfering RNAs (siRNAs), drug molecules or nanoparticles, thereby reducing toxic and side effects (Bagalkot et al., 2006; Farokhzad et al., 2006; McNamara et al., 2006; Han et al., 2020). Atabi et al. (2017) prepared DNA aptamer-linked myristate-chitosan nanogels for targeted prostate cancer therapy. Myristate-chitosan nanogels (MCS) were linked to a selective ssDNA aptamer capable of detecting androgen-sensitive human prostate adenocarcinoma cells, known as LNCap. The resulting nanogel complex was loaded with Doxorubicin (DOX) to form Apt-MCSDOX complex for targeted drug delivery against the prostate cancer cells. LNCaP cells were treated with Apt-MCSDOX complex. The Apt-MCSDOX complex was cytotoxic to the LNCaP cells. The results showed the potential of aptamer–based nanogels in targeted prostate cancer therapy.

#### 2.5.6 Nanogel formulations for bladder cancer therapy

Bladder cancer is the 12th -most common type of cancer globally (Wild et al., 2020). Nanogels have been proposed as promising formulation strategy for bladder cancer targeted therapy (Guo et al., 2017).

##### 2.5.6.1 Penetrable polypeptide nanogel for efficient drug delivery to orthotopic bladder cancer

There is sub-optimal absorption of drugs used in the treatment of bladder cancer due to the poor penetration of the drugs through the bladder wall. The small quantity of drugs which are able to penetrate the bladder walls are often excreted rapidly as urine (Guo et al., 2017). 10-Hydroxycamptothecin (HCPT) is derived from camptothecin extracted from the Chinese plant *Camptotheca acuminata* (Nyssaceae) and exhibits anti-cancer activity (Zhang et al., 2000). Guo et al. (2017) synthesized a reduction-responsive disulfide-core-cross-linked polypeptide nanogel of poly (l-lysine)–poly (l-phenylalanine-*co*-l-cystine) (PLL–P (LP-*co*-LC)) containing 10-Hydroxycamtothecin (HCPT). The nanogel formulation penetrated the bladder walls, and released optimal concentrations of HCPT into bladder cancer cells. The HCPT nanogel was also retained longer in the bladder walls and had fewer side effects when compared with free HCPT. This proves that nanogels formulations of anti-cancer drugs have the potential to improve penetration of drugs through the bladder wall and ensure optimal drug concentrations in bladder cancer therapy.

#### 2.5.7 Nanogel formulations for glioma therapy

Cancer of the brain and central nervous system (Glioma) is the 17th most common cancer type. Although scientists are still in the process of understanding the mechanism of malignant glioma formation, it is believed to be linked to the immune system (Wild et al., 2020). Nanogels have been confirmed to be efficient in crossing the blood-brain barrier for optimal targeted brain cancer therapies (Stawicki et al., 2021). Furthermore, nanoparticles can be designed by incorporation of ligands to trigger either carrier-mediated transport or receptor-mediated transcytosis across the blood brain barrier (Saraiva et al., 2016; Hersh et al., 2022). Singh et al. (2021) developed nanogels functionalized with diphtheria toxin receptor ligand which enabled receptor-mediated transcytosis (transcellular transport) of nanogels through the blood brain barrier into the tumor cells. Song et al. (2021) developed nanogels using modified peptide angiopep-2 which led to improved infiltration of doxorubicin across the blood brain barrier.

##### 2.5.7.1 Lipid-based carbopol-gellan gum nanogel for glioma therapy

Terifunomide is currently being investigated for its potential therapeutic efficacy against glioma (Gadhave et al., 2021). Gadhave et al. (2021) formulated intra-nasal teriflunomide (TFM)- loaded nanogels to evaluate its anti-cancer efficacy. They used a variety of materials including gellan gum, carbopol 974P and a blend of lipids in the nanogel formulation. The results of anti-cancer efficacy tests revealed that the nanogels formulations had a 2-fold higher efficacy than other formulations. This confirms the possibility of efficacious treatment of glioma using intra-nasal nanogels of anti-cancer drugs for cancer patients.

#### 2.5.8 Nanogel formulations for therapy in multiple cancer types

##### 2.5.8.1 Transdermal curcumin nanogel for squamous cell carcinoma therapy

Squamous cell carcinoma (SCC) is an epithelial malignancy that occurs in various organs and tissues covered with squamous epithelium. The majority of SCC cases are reported to occur in non-melanoma skin cancer, head and neck cancer, esophageal cancer, and non-small cell lung cancer (Yan et al., 2011). In the last 30 years, progress has been made in the diagnosis and treatment of SCC, however, the survival rate of SCC is still increasing due to lack of reliable early diagnostic cancer biomarkers and limited efficient targeted therapies (Noguti et al., 2012). Ganesh et al. (2019) formulated transdermal curcumin nanogels to enhance the solubility, and permeability of curcumin. They selected the oil, surfactant and co-surfactants used in the nanogel formation due to their physicochemical properties. The results of the characterization and evaluation of the nanogels revealed they had good viscosity. They also exhibited optimal transdermal permeation with a higher drug release and less toxicity than conventional curcumin formulations.

#### 2.5.9 Nanogel formulations for ovarian cancer therapy

Ovarian cancer is frequently aggressive and is generally detected at a late stage. It is the eighth most common cause of cancer death in women worldwide (Wild et al., 2020).

##### 2.5.9.1 Nanogel discs for adjuvant ovarian cancer therapy

Cho et al. (2019) demonstrated the application of 3D printing technology in fabricating nanogel discs loaded with paclitaxel and rapamycin for adjunct ovarian cancer therapy. The nanogels were stable during storage. *In vivo* evaluation showed that the nanogel discs were successful in the peritoneal delivery of paclitaxel and rapamycin in ES-2-luc ovarian-cancer-bearing xenograft mice.

##### 2.5.9.2 Dendrimer-decorated nanogels for ovarian cancer therapy

Li et al. (2021) combined poly (amido amine) (PAMAM), Poly (*N*-vinylcaprolactam) (PVCL), and glycidyl methacrylate (GMA) to develop dendrimer-decorated PVCL-GMA nanogels (NGs) for ovarian cancer therapy. The unique features of the polymers enabled manifestations of dual thermal and pH-responsive behaviors, which enhanced targeted drug delivery with minimal adverse effects.

##### 2.5.9.3 Hyaluronic acid nanogels for ovarian cancer therapy

Limiti et al. (2022) designed hyaluronic acid nanogels for ovarian cancer therapy. They applied “a novel emulsion-based strategy” to produce nanogels by chemically cross–linking hyaluronic acid (HA) and polyethyleneimine (PEI). Their strategy resulted in the optimization of the physico-chemical interactions between HA and PEI which led to the formation of a stable emulsion without the use of surfactants. The dispersed phases of the emulsion were aqueous solutions of HA and PEI while the continuous phase was an organic solvent. The HA-PEI nanogels obtained were evaluated and exhibited potential therapeutic efficacy for ovarian cancer therapy. They were stable and showed better sustained release profile when compared with the free drug. An *in vitro* assay with a CD44 blocking/neutralizing antibody revealed that “hyaluronan receptor was involved in the nanogel internalization process.”

##### 2.5.9.4 Cisplatin-loaded nanogel for peritoneally disseminated ovarian cancer

Yamaguchi et al. (2021) designed a cisplatin (CDDP)-loaded nanogel for peritoneally disseminated ovarian cancer to ensure adequate exposure of ovarian cancer cells to optimum concentrations of cisplatin. They developed “an alginate (AL)-based hybrid system in which a CDDP-loaded AL nanogel (AL/CDDP-nanogel) was encapsulated in an injectable AL-hydrogel cross-linked with calcium ions.” This matrix prevented the rapid clearance of CDDP and facilitated a sustained release of CDDP from the nanogel hybrid for over 1 week. The nanogel exhibited therapeutic efficacy against ovarian cancer *in vivo* and is a promising therapy for ovarian cancer.

## 3 Clinical trials of nanogels as targeted drug delivery systems for cancer therapy

### 3.1 Clinical trials on OncoGel formulation

Proteins and drugs encapsulated in nanogels are protected from degradation caused by unfavorable environmental factors. ReGel™ is a thermo-sensitive triblock copolymer based delivery system with a PLGA–PEG–PLGA structure. OncoGel, a formulation of ReGel™ encapsulating paclitaxel, has been investigated for use in cancer therapy. The phase 1 study by Vukelja et al. (2007) was designed to assess the toxicity and efficacy of OncoGel for treatment of solid tumors. Four doses from 0.06 to 2.0 mg paclitaxel/cm^3^ tumor volume of OncoGel was injected right into 18 superficial solid tumors of 16 cancer patients with no option of curative therapy. OncoGel injections were generally well tolerated. Out of the 14 patients who were assessed for disease progression, six had stable disease and eight had progressing disease. The drug remained localized at the tumor site thereby minimizing systemic effects. In the phase II dose escalation study, eleven esophageal cancer patients who were in advanced state of the disease and eligible for palliative external-beam radiotherapy (RT) were enrolled. Using standard endoscopic method, OncoGel was administered into the primary tumors, and RT initiated afterwards. From the result of the study, a combination therapy of OncoGel and RT was well tolerated in patients with inoperable esophageal cancer. The release of paclitaxel from the formulation was sustained and systemic exposure was minimal. Improvement in symptoms, reduction in tumor size and negative esophageal biopsies were seen in these patients (Duvall et al., 2009). Therefore, the use of OncoGel together with RT reduced tumor burden in these patients. Although some non-clinical studies on combinational therapy of OncoGel with surgery and radiation therapy have been carried out as reported in a review by Elstad and Fowers (Elstad and Fowers, 2009), this therapy should be further investigated in human.

### 3.2 Clinical trials on cholesteryl pullulan (CHP) nanogels

Cholesteryl pullulan (CHP) based vaccine candidates are the most promising applications of nanogels in cancer therapy. Cancer vaccines based on CHP nanogels have been in clinical trials since the late 2 decades with promising results. The preparation of CHP nanogel has been discussed in Section 2.3.1.3. CHP serves as a vehicle to protect and transport of antigen to antigen-presenting cells of the immune system. The commonly encapsulated antigens for cancer vaccines using CHP nanogels are Human epidermal growth factor receptor type 2 (HER2), New York esophageal squamous cell carcinoma 1 (NY-ES0-1), and melanoma related antigen A (MAGE-A4) (Kageyama et al., 2008; Ueda et al., 2018; Ishihara et al., 2020; Ishikawa et al., 2021).

#### 3.2.1 Clinical trials on CHP-HER2 vaccine

HER2 is a growth-promoting protein on breast cells. HER2 protein overexpression has been found in several tumors including breast, esophageal, lung, cervical, bladder, pancreatic, ovarian, and stomach cancers. In a clinical trial to investigate the safety as well as specific immune responses to HER2, nine HER2-expressing cancer patients were administered 300 μg of the CHP-HER2 vaccine subcutaneously thrice at interval of 2 weeks. The vaccine was well tolerated with only grade 1 reaction at injection sites. There was induction of specific CD8^+^ and CD4^+^ immune responses in five patients (Kitano et al., 2006).

In another similar clinical trial by Kageyama et al [158], fifteen patients with HER2-expressing tumor were enrolled to investigate the safety and specific cellular and humoral immunological responses. Adjuvants were added to the antigen and their ability to enhance the immune response was assessed. The study was split into two phases. For the first phase of the study, nine patients were injected with the vaccine subcutaneously biweekly. After receiving the fourth dose of the vaccine, the patients were given one of these two adjuvants–human granulocyte-macrophage colony-stimulating factor (GM-CSF) or OK-432. OK-432 is a low-virulence *Streptococcus pyogenes* strain that has been killed and lyophilized. In the second phase of the study, a combination of CHP-HER2 vaccine and GM-CSF was administered to six patients. The vaccination with CHP-HER2 vaccine induced the production of IgG antibodies in 14 formerly seronegative patients. The findings show that CHP-HER2 vaccine elicited HER2-specific humoral responses in patients with HER2-expressing malignancies, and that GM-CSF appears to speed up these responses. Despite the fact that the CHP-HER2 vaccination was successful in enhancing the induction of HER2 specific antibodies, there was no evidence of tumor regression in any of the participants.

#### 3.2.2 Clinical trials on CHP-NY-ESO-1 vaccine

NY-ESO-1 is a cancer-testis antigen with restricted expression in germ cells and placental cells but frequently occur in multiple tumors like melanoma and carcinoma of lung, esophageal, liver, and prostate. It is a highly immunogenic antigen. However, due to the limited stability of NY-ESO-1 in aqueous solution, clinical applications will require an effective delivery system. Cholesterol-bearing hydrophobized pullulan (CHP) nanogels have been used to encapsulate NY-ESO-1 (CHP-NY-ESO-1) antigen and the safety and effectiveness tested in clinical trials. Kawabata et al. (2007) investigated the humoral immune response of nine patients vaccinated with CHP-NY-ESO-1. CHP-NY-ESO-1 vaccination resulted in the induction or amplification of NY-ESO-1 antibody responses in all nine subjects. Antibodies produced after vaccination recognized five epitopes in the NY-ESO-1 protein molecule. According to their findings, vaccination with CHP-NY-ESO-1 can produce robust humoral immune responses against NY-ESO-1 antigen in cancer patients.

In another clinical trial, biweekly vaccination with CHP-NY-ESO-1 induced antibody production in all the 9 patients (4 esophageal cancers, 4 prostate cancers and one malignant melanoma patients) enrolled in the study. This finding is in agreement with previous clinical trial and confirms the efficacy of CHP-NY-ESO-1 in eliciting humoral immune response. An enhancement in the CD4 T cell and CD8 T cell response was also seen in seven out of the nine patients. Unlike the CHP-HER2 vaccination where a truncated protein was used, tumor regression and improvement in symptoms were observed in four patients (3 out of 4 esophageal cancer patients and a malignant melanoma patient) vaccinated with full length NY-ESO-1 antigen. There was stabilization of prostate-specific antigen (PSA) values in 3 out of the 4 prostate cancer patients during the course of vaccination. The findings of this study suggest that using whole protein in cancer vaccines could help prevent cancers from eluding the immune response (Uenaka et al., 2007).

There was a follow-up study on the patient with stage IV malignant melanoma. Blisters appeared at the lesion sites on day 25. The skin sample revealed a significant number of apoptotic melanoma cells expressing NY-ESO-1. Due to the presence of multiple metastases, the patient died on day 48. Antibody production was observed after the second dose of the vaccine, and the titre value rose with subsequent vaccinations. There was induction of NY-ESO-1-specific CD4 and CD8 T cell responses in this patient. Like the CHP-NY-ESO-1 vaccination generated specific humoral and cellular immune responses against NY-ESO-1 in melanoma patients. Immune escape could be explained by the presence of regulatory T cells, immune regulatory macrophages, and cytokines at the local tumor sites in this patient (Tsuji et al., 2008).

In additional phase I clinical study, 8 esophageal cancer patients, including 4 from the previous study and 4 newly enrolled patients, were evaluated. Patients were injected subcutaneously at biweekly intervals with CHP-NY-ESO-1. Antibody induction was observed in seven patients while CD4 and CD8 T-cell responses were observed in 7 and 6 patients respectively. No significant adverse events were observed. The induction of NY-ESO-1 immunity and some positive clinical responses were observed in esophageal cancer patients. Notwithstanding, the tumors grew even after vaccination (Wada et al., 2008).

A clinical trial using a combination of CHP-NY-ESO-1 and CHP-HER2 was carried out after the CHP-NY-ESO-1 vaccination showed encouraging outcomes. The CHP-NY-ESO-1 and CHP-HER2 combination vaccines were given to eight esophageal cancer patients with the adjuvant OK-432. In the combination vaccine research, responses to NY-ESO-1 were comparable to those of the single vaccine while there was reduced antibody response to HER2 in the combination vaccinations. Antibodies to OK-432 were not boosted. The combination vaccine was well tolerated, with just minor side effects, indicating feasibility and safety of targeting both NY-ESO-1 and HER2 using one vaccine (Aoki et al., 2009).

Kawada et al. (2012) demonstrated that heteroclite serological responses could be used as indicator of the overall immune response against NY-ESO-1 antigen in a phase 1 clinical trial. 13 patients were vaccinated with CHP-NY-ESO-1 vaccine and there was efficient production of antibodies against NY-ESO-1, as well as induction of CD4 and CD8 T cell responses. Heteroclite serological response analysis was carried out on these patients after vaccination. Out of nine patients that demonstrated specific humoral responses, eight showed humoral responses against at least 1 of 11 tumor antigens.

The safety as well as the immune and clinical outcomes of patients who received 100 μg or 200 μg of NY-ESO-1 protein complexed with CHP were evaluated in a clinical trial. Twenty-five patients with advanced/metastatic esophageal cancer were vaccinated subcutaneously with either 100 μg or 200 μg of NY-ESO-1 protein encapsulated in CHP nanogel. Thirteen patients were administered 100 μg of the vaccine while twelve patients received 200 μg, and the median doses were 8 and 9.5 doses, respectively. There were no major side effects associated with the vaccination. In the group that received 100 μg of the antigen, 5 out of 10 patients that were seronegative before the treatment showed antibody response, while antibody titers were raised in 2 patients that were seropositive before the vaccination. All five pre-seronegative patients in the cohort that received 200 μg turned positive, and there was elevation of antibody titer in all seven pre-seropositive patients. There was no evidence of tumor shrinkage. The patients in the 200 μg cohort, even patients who had more cancer burden or were unresponsive to previous treatment measures, demonstrated better survival than patients in the 100 μg cohort. The safety and immunogenicity of CHP-NY-ESO-1 vaccine were confirmed with this clinical study. The immune responses more effectively and suggested better survival benefits (Kageyama et al., 2013).

In a recent randomized phase II clinical trial of CHP-NY-ESO-1to determine the clinical efficacy of the vaccine, 54 NY-ESO-1-expressing esophageal squamous cell carcinoma (ESCC). Patients were enrolled. Fifteen doses of the vaccine were subcutaneously given at 2 or 4-week interval for a duration of 12 months. There were no differences between the treated group and the control group in terms of disease free survival and overall survival. The clinical trial revealed that CHP-NY-ESO-1 vaccine alone did not display clinical efficacy compared to the control (Kageyama et al., 2019).

Based on the above result, MIS416, a micro particle adjuvant obtained from *Propionibacterium acnes*, which have shown promising result in mouse model (Girvan et al., 2011) was used together with CHP-NY-ESO-1 in a clinical trial. The vaccination schedule was made up of treatment phase followed by maintenance phase. In the treatment phase, six doses of CHP-NY-ESO-1/MIS416 was given to four groups of patients with NY-ESO-1-expressing refractory solid tumors at doses of 100/200, 200/200, 200/400 or 200/600 μg/μg biweekly. The maintenance phase followed afterwards with the patients being vaccinated once every 4 weeks. This vaccination continued until there was evidence of disease progression or development of unacceptable toxicity. There were no grade 4–5 adverse effects. Eight of the patients had a stable disease (SD). An increase in MIS416 dose did not result in an increase in antibody or humoral responses. A preclinical investigation carried out revealed that vaccination with CHP-NY-ESO-1 and MIS416 together with anti-PD-1 monoclonal antibody resulted in considerable tumor regression (Ishihara et al., 2020). This new combination therapy appears to be a good step forward.

Poly-ICLC is a toll-like receptor (TLR)-3 agonist and it was investigated for use as adjuvant with CHP-NY-ESO-1 vaccine. In a recent clinical trial, poly-ICLC was used together with CHP-NY-ESO-1 in patients diagnosed with recurrent or advanced esophageal cancer. A total of six vaccinations at a dose of 200/0.5 or 200/1.0 (μg/mg) of CHP-NY-ESO-1/poly-ICLC was carried out biweekly. The tumor response, as well as the safety and immune response, were studied. The injection site skin reaction was reported as the most prevalent adverse event (AE). There were no drug-related AEs of grade 3 or higher. The immune responses were not dose dependent; and with a median of 2.5 vaccinations, all patients developed antibody responses. Both the group that received CHP-NY-ESO-1 alone and the group that received the poly-ICLC combination showed humoral responses, however the antibody titers in the combination group were higher. There was no tumor response. Like the previous non-clinical study with anti-PD-1 antibody, CHP-NY-ESO-1/poly-ICLC/anti-PD-1 combination vaccine inhibited the growth of tumors expressing NY-ESO-1 antigen in a mouse model (Ishikawa et al., 2021). In human trials, combining the vaccination with PD-1 blockade appears to be promising.

#### 3.2.3 Clinical trials on CHP-MAGE-A4 vaccine

CHP-MAGE-A4 is a melanoma vaccine candidate that has the MAGE-A4 antigen encapsulated in cholesteryl pullulan nanoparticles. The MAGE-A4 protein is not found in normal tissues but expressed in many cancers, and it triggers both antibody production and cell mediated immune responses (Tahara and Akiyoshi, 2015). In a phase I clinical trial, the safety, immune and clinical responses of CHP-MAGE-A4 were studied, as well as the prognostic factors for vaccination. A total of six doses of 300 µg MAGE-A4 was given subcutaneously to 20 patients with advanced esophageal, stomach, or lung cancer. The vaccine was found to be well tolerated by the patients. One immunization round was completed by 15 of the 20 patients, and two patients had stable disease (SD). Four patients who expressed high levels of MAGE-A4 or MHC class I antigen on tumor cells had considerably higher overall survival than those whose tumor cells expressed low levels. Three and six patients, respectively, had CD4 and CD8T cell responses. The researchers suggested that MAGE-A4 and MHC class I expression in tumor tissue, as well as the activation of a MAGE-A4-specific immune response following vaccination, could be useful prognostic markers for MAGE-A4 vaccine recipients (Saito et al., 2014).

In another CHP-MAGE-A4 vaccine trial, 9 patients were vaccinated biweekly up to 6 times to assess the safety of administering CHP-MAGE-A4 with and without OK 432 and to assess the specific humoral immune response. 100 μg CHP-MAGE-A4, 300 μg CHP-MAGE-A4, or 300 μg CHP-MAGE-A4 + 0.5 clinical units of OK-432 were given to each group of three patients. There were no serious side effects from the vaccines. Four out of nine individuals were found to have stable disease. Total immunoglobulin (Ig) G titers against MAGE A4 increased in 7 of 9 individuals. IgG4 and IgE were found in 4/7 of the patients following repeated vaccination. It was found that frequent immunizations activate a Th2 dominant state in cancer patients. The authors linked the unfavorable clinical outcomes such as disease progression and the emergence of a new relapse lesion to Th2 conversion. The detection of a time dependent IgG subclass and IgE antibody production during vaccination could be a valuable surrogate marker suggesting a possibly negative changes in the immunological microenvironment for a successful antitumor immune response in cancer patients according to the researchers (Kyogoku et al., 2016).

A clinical trial by Kengo Miyauchi et al. (2016) assessed the clinical relevance of antigen spreading pattern as a surrogate index of patient survival in CHP-MAGE-A4-vaccinated patients. 12 individuals who had received more than five vaccines with 300 μg of CHP-MAGE-A4 and 0.5 Klinische Einheit of OK-432 were assessed. After five vaccinations, 8, 6, and 5 patients developed antibody responses to MAGE-A4, NY-ESO-1 and MAGE-A3, respectively. According to the researchers, the pattern of antigen spread could be a reflection of tumor shrinking as a result of treatment.

Still on the search for good immune response markers, a dose of 300 μg of CHP-MAGE-A4 vaccine and 0.5 Klinische Einheit of OK-432 was administered to sixteen patients to investigate the effectiveness of predicting immune response using the percentage of CD4^+^CD25 + Foxp3+regulatory T cells (Tregs). Low Treg ratios both before and after vaccination were associated with a good prognosis, and a low Treg/CD4 lymphocyte ratio 7-week after the initial vaccination was correlated with a better prognosis. It was concluded that the Treg ratio following vaccination could be useful for predicting patient prognosis (Wada et al., 2018).

The safety, immunological responses, and clinical outcomes of patients who received the CHP-MAGE-A4 vaccine were investigated in a recent clinical study. Twenty-two patients who participated in the study had advanced or metastatic cancer and were given either 100 μg or 300 μg of CHP-MAGE-A4 subcutaneously with a median of seven doses (Ueda et al., 2018). There were no major side effects associated with the vaccination. Two of the seven patients (29%) who received the 100 μg dose had immunological responses, compared to three of the fourteen (21%) who received the 300 μg dose and there were no differences in survival between patients who received the 100 and 300 μg dosages. The survival time was considerably reduced in 16 individuals with esophageal or head/neck squamous cell carcinoma who had NY-ESO-1-co-expressing tumors. Patients who had a lot of pre-existing antibody responses to NY-ESO-1 had a worse prognosis than those who didn’t have any. As a result, whether or not to include NY-ESO-1-expressing tumors in MAGE-A4 vaccination clinical trials is a crucial issue to address. One strategy for overcoming the poor prognosis would be to combine MAGE-A4 and NY-ESO-1 antigens in a vaccine.

## 4 Future prospects of nanogels as target drug delivery systems in cancer therapy

Nanogels hold the promise for unlocking the door to efficient target drug delivery to cancer cells and tissues. Cancer chemotherapy involves the interplay of a lot of factors ranging from drug related factors to patient-related factors. In all, the *in vitro* and *in vivo* stability of the dosage form is extremely important to ensure the dosage form reaches the target site intact. Nanogels have proven to be a very stable dosage form that can provide the needed payload to cancerous tissues through the main delivery routes of medicines for cancer–the intravenous route. However, considering the versatility of nanogels, topical formulations will likely be useful in localized skin tumors as their permeability can be optimized through appropriate engineering. Biomimetic hydrogels are expected to play a very significant role in intracellular drug delivery as they are expected to be biocompatible, and would evade the reticuloendothelial system and morph nuclear phagocytic system leading to prolonged *in vivo* circulation time. It is therefore the onus upon drug delivery scientist to focus on these new classes of nanogels without neglecting the use of natural polymers, which have traditionally been recognized as safe, biocompatible and biodegradable. Another aspect of nanogels that would attract the attention of formulation scientists in the next decade is the use of polymer hybrids or molecular blends consisting of mixtures of synthetic and natural polymers. The synthetic polymers would provide more functionality, while natural polymers would positively influence the biocompatibility and biodegradability. Natural polymers also do not produce toxic degradation products unlike some synthetic polymers and would serve as a very desirable candidate for implant delivery system for anticancer drugs. Nanogels can be implanted into the tumor sites and drug release will occur as predicted by the engineering strategies applied during formulation. This method of delivery will reduce drug migration to unwanted sites and thereby drastically reduce off-target toxicities associated with systemic delivery of some anticancer drugs.

## 5 Conclusion

Nanogel formulations of anti-cancer drugs have extended the horizon for what might be one of the most important revolutions in targeted cancer therapies. Preliminary research on the potential applications of nanogels in targeted cancer therapy has shown great promise for the future. However, there is a need to standardize processes with a good manufacturing practice guideline for nanogel formulations. It is also important to scale-up nanogels research for targeted cancer therapy to expand current knowledge of nanogels and confirm their safety profile.

The results of multiple clinical trials using CHP-based anticancer vaccines showed that the vaccines were safe after repeated subcutaneous injection and that they were effective in generating both antigen-specific CD4^+^ and CD8^+^ T-cell responses as well as humoral immunity. Although the production of antigen-specific T-cell responses following vaccination is encouraging, more research is needed to properly comprehend CHP’s true potential or effectiveness in cancer vaccination. Despite a large number of clinical trials, the clinical efficacy of nanogel in cancer vaccines has been found to be insufficient, and the intended clinical outcome of tumor regression has not been detected in a significant number of clinical trials. Understanding the precise immunological pathways of immune response generation is critical for improving the efficacy of nanogel based cancer vaccines. Identification of more immunological biomarkers that could allow for a more precise assessment of the clinical outcome to cancer vaccination will surely be advantageous in this context. The overall prospect of nanogels as target delivery system for anticancer drugs remains high and start-up pharma companies are expected to utilize the opportunity offered by nanogels to advance anticancer drugs translational research.

## Data Availability

The original contributions presented in the study are included in the article/Supplementary Material, further inquiries can be directed to the corresponding authors.
